# Suppression of *RNA-Dependent RNA Polymerase* *6* Favors the Accumulation of Potato Spindle Tuber Viroid in *Nicotiana Benthamiana*

**DOI:** 10.3390/v11040345

**Published:** 2019-04-14

**Authors:** Charith Raj Adkar-Purushothama, Jean-Pierre Perreault

**Affiliations:** RNA Group/Groupe ARN, Département de Biochimie, Faculté de médecine des sciences de la santé, Pavillon de Recherche Appliquée au Cancer, Université de Sherbrooke, 3201 rue Jean-Mignault, Sherbrooke, QC J1E 4K8, Canada; Charith.adkar@usherbrooke.ca

**Keywords:** viroid, RNA-dependent RNA polymerase 6, RNA silencing, Potato spindle tuber viroid, PSTVd

## Abstract

To date, two plant genes encoding *RNA-dependent RNA polymerases* (RdRs) that play major roles in the defense against RNA viruses have been identified: (i) RdR1, which is responsible for the viral small RNAs (vsRNAs) found in virus-infected plants, and, (ii) RdR6, which acts as a surrogate in the absence of RdR1. In this study, the role of RdR6 in the defense against viroid infection was examined by knock-down of RdR6 followed by potato spindle tuber viroid (PSTVd) infection. The suppression of RdR6 expression increased the plant’s growth, as was illustrated by the plant’s increased height. PSTVd infection of RdR6 compromised plants resulted in an approximately three-fold increase in the accumulation of viroid RNA as compared to that seen in control plants. Additionally, RNA gel blot assay revealed an increase in the number of viroids derived small RNAs in RdR6 suppressed plants as compared to control plants. These data provide a direct correlation between RdR6 and viroid accumulation and indicate the role of RDR6 in the plant’s susceptibility to viroid infection.

## 1. Introduction

Viroids are non-coding RNA pathogens which exclusively infect plants. They are small (246 to 401 nucleotides), highly structured, single-stranded, circular RNA molecules [1]. These species are broadly classified into two families, the *Pospiviroidae* and the *Avsunviroidae* [2]. The members of the family *Pospiviroidae*, (the type species being potato spindle tuber viroid (PSTVd)), contain five structural/functional domains, a central conserved region and replicate in the nucleus of infected cells through an asymmetric rolling circle mechanism. In contrast, the members of the family *Avsunviroidae* (the type species being avocado sunblotch viroid (ASBVd)), do not have any structural/functional domains, nor a central conserved region. However, they do exhibit self-cleavage activity through a hammerhead ribozyme, and they replicate via a symmetric rolling circle mechanism in the chloroplasts of infected cells [3].

Depending on the host plant, and on the type of viroid species and/or variant, upon infection, a viroid may induce a wide array of symptoms in the host plants including stunting, leaf epinasty and leaf distortion [3,4,5]. Due to the internal base-paired structure of mature viroids, and to the formation of double-stranded RNA during viroid replication, viroids are both inducers and targets of RNA silencing [6]. The accumulation of viroid-derived small RNAs (vd-sRNA) of 21 to 24 nucleotides in length has been detected in viroid-infected plants, supporting the hypothesis that viroid infection triggers the RNA silencing machinery [7,8]. This hypothesis is supported by previous studies with different host-viroid combinations, by the RNA inference-mediated down-regulation of a host’s mRNA by the direct interaction of the vd-sRNA and by the possible involvement of either secondary small RNAs or phased secondary small RNAs (phasiRNAs) that has been discussed by several groups [9,10,11,12,13,14,15].

RNA interference is an RNA sequence-specific defense mechanism in higher organisms that is triggered by dsRNAs, such as that of viruses. These dsRNAs are processed into 21- to 24-nucleotide long small RNAs (sRNA), known as small interfering RNAs (siRNAs), by the *RNase III*-type ribonucleases (DICER or DICER-LIKE). The RNA-induced silencing complex recruits these siRNAs to direct the degradation of complementary RNAs [16,17]. In plants, *RNA-dependent RNA polymerases* (RdR, previously, silencing defective (SDE)1/suppressor of gene silencing (SGS)2) have been shown to be essential for virus-induced post-transcriptional gene silencing [18,19,20]. The RdRs are also known to be involved in the production of secondary siRNAs, such as phasiRNAs, that play a role in the amplification of the initial RNA silencing reaction [21,22,23]. The latter is a transitivity phenomenon. Certain viruses counteract this host defense mechanism by encoding suppressors of RNA silencing in order to ensure their successful invasion of the host plant [24,25]. An RdR encoding gene, specifically the RdR6 of *Arabidopsis*, is a plant homolog of the *Neurospora crass* QDE-1. The mutation of the RdR6 in *Arabidopsis* showed that it was specific for the PTGS induced by transgenes rather than that induced by viruses. That said, the role of RdR6 is to synthesize a dsRNA, the initiator of post-transcriptional gene silencing [19]. The RdR6 mutant lines of *Arabidopsis* inoculated with either the crucifer strain of tobacco mosaic virus, the tobacco rattle virus or the turnip crinkle virus did not exhibit any effects on accumulation levels of these viral RNAs [18,19].

In plants, six *RdRs* have been isolated: RDR1, RdR2, RdR3, RdR4, RdR5 and RdR6 [26]. Microarray experiments revealed different developmental and stress-responsive patterns for these six RdRs in *Arabidopsis*. RDR1 is highly expressed in old leaves and the inflorescence apex. RdR3 and RdR4 are predominant in the inflorescence apex, while the other RdRs are consistently expressed during development. Further, more RdR5 is found in dry seeds, while the RdR6 gene is found to respond the most to stress [27]. By comparing the tomato genome sequence data (http://solgenomics.net/) with that of the known six *Arabidopsis* RdRs genes, six homologous genes encoding RdR in tomato were detected [28]. In addition, PSTVd infection of tomato plants is known to induce RdR1, and this gene has been implicated in the anti-viroid defense mechanism [29]. In *Nicotiana benthamiana* (*N. benthamiana*) it has been shown that RdR1 might contribute to both the salicylic acid-mediated antiviral defense and suppressing, as well as to the RDR6-mediated antiviral RNA silencing [30]. Additionally, transgenic *N. benthamiana* expressing a functional *RdR1* increased resistance against tobacco mosaic virus but not against cucumber mosaic virus or potato virus *X* indicating the differential anti-virus defense against different groups of viruses [31]. Previously, the role of RdR6 in both viroid-induced symptom expression and the accumulation of vd-sRNA in *Nicotiana benthamiana* (*N. benthamiana*) was demonstrated using the hop stunt viroid (HSVd) [32]. Gómez et al., [32] analyzed a symptomatic transgenic line of *N. benthamiana* that expressed and processed dimeric forms of hop stunt viroid under both different growth temperature conditions and in grafting assays with the *rdr6i-Nb* line in which the RDR6 was constitutively silenced. Though the scion of the *rdr6i-Nb* plants exhibited the accumulation of hop stunt viroid, they were devoid of symptoms. In contrast, the stocks were symptomatic. In a separate study, it was shown that RdR6 delays the accumulation and inhibits the meristem invasion of PSTVd in *N. benthamiana* plants [33]. Furthermore, it has been previously been shown that RdR6 mutant plants displayed enhanced susceptibility to cucumber mosaic virus infection [18]. Hence, in order to study the role of RdR6 in the host’s susceptibility to PSTVd infection, both biotechnology and molecular biology assays were performed in *N. benthamiana* plants.

## 2. Materials and Methods

### 2.1. Construction of the PSTVd cDNA, Transcription, and Plasmids for the VIGS

The dimeric construct of PSTVd-RG1 (GenBank Acc. No. U23058) was used to synthesize infectious dimeric transcripts as described previously [11]. The pTRV1 and pTRV2 vectors for the virus-induced gene silencing (VIGS) assay were procured from Arabidopsis Biological Resource Centre (Ohio, USA). In order to produce the pTRV2 derivative targeting the *RdR6* mRNA, 400 base pairs of the tomato *RdR6* gene (GenBank Acc. No. NM_001279276) was commercially synthesized with *Xba*I and *Bam*HI restriction sites at the ends (GenScript, Piscataway, NJ, USA). This DNA string of tomato RdR6 gene showed 90% sequence similarity with the RdR6 gene of *N. benthamiana* (GenBank Acc. No. AY722008). This DNA was then ligated into the pGEM-T vector (Promega, Madison, WI, USA) after adding -A overhangs, and the resulting plasmids were then digested with the restriction endonucleases *Xba*I and *Bam*HI. The digested products were ligated into the same restriction sites of the binary vector pTRV2. The resulting binary vector was named pTRV2-RdR6. The plasmids were then transformed into the *Agrobacterium tumefaciens* strain GV3101 as previously described [34]. The pTRV2 derivative targeting the *Phytoene desaturase* (PDS) mRNA (pTRV2-PDS) previously described was used as a positive control for the VIGS experiments [35].

### 2.2. Plant Material and Growing Conditions

The *N. benthamiana* used for the viroid inoculation assays were grown in a growth chamber at 25 °C with 16 h of light and 8 h of darkness. After the development of the primary leaves, the plants were agro-infiltrated using a 1 mL needleless syringe and were then grown at 23 °C with 16 h of light and 8 h of darkness [11]. After the observation of bleaching in the plants agroinfiltrated with pTRV2-PDS, the plants agroinfiltrated with either the pTRV2-empty vector (pTRV2-EV) or the pTRV2-RdR6 were mechanically inoculated with 1 μg of PSTVd-RG1 RNA transcripts and were incubated described as above.

### 2.3. RNA Extraction and RT-qPCR

Total RNA was extracted from the leaf samples using the *mir*Vana^TM^ miRNA isolation kit (Ambion, Austin, TX, USA), and complementary DNA (cDNA) was synthesized as described previously [11]. For the evaluation of the suppression of both RdR6 and PSTVd accumulation, 10 ng of cDNA were used for quantitative polymerase chain reaction (qPCR) in the presence of the RdR6-F/RdR6-R and PSTV-231F/PSTV-296R primer pairs, respectively [36,37]. Reverse transcription-qPCR (RT-qPCR) data were also obtained for three housekeeping genes, specifically the 5.8S, 18S, and 25S rRNAs, and were used for normalization. Each experiment was performed at least three times with true biological replicates, and all qPCR runs included a negative control. The qBASE framework was used to calculate the relative expression levels [38].

### 2.4. Riboprobe Preparation and *RNA* Gel Blot Analysis

In order to detect PSTVd in the RNA samples extracted from leaves, dimeric (−) PSTVd riboprobe was used. For the detection of RdR6, a portion of the RdR6 from tomato (*Solanum lycopersicum* cv. Rutgers) was amplified and cloned into the pBluescript KS+ vector. The expression of the 5S rRNA was used as the loading control for the RNA gel blot assay. Specifically, full-length 5S rRNA, which was previously amplified from tomato cv Rutgers and cloned into pBluescript KS+ vector, was used for riboprobe preparation [39]. In order to prepare the riboprobes, either the T3 or the T7 MAXIscript kit (Ambion) was used after linearization of the plasmid with the appropriate restriction endonuclease. For the RNA gel blot hybridizations, total RNA samples extracted from 21 days post-infection (dpi) plants were denatured at 65 °C for 10 min using 2.5 volumes of sample buffer (50% formamide, 2.2 M formaldehyde (37%), and 1× MOPS), and were then separated on 1.2% agarose-formaldehyde gels containing 1× MOPS buffer. For the detection of both the PSTVd accumulation and the RdR6 expression, 2.0 μg and 5.0 μg of total RNA were used, respectively. The Northern gel blot hybridizations were performed as described previously [11].

For the detection of PSTVd-sRNA, of the total RNA (10.0 μg) extracted from 21-dpi plants were transferred to Hybond-XL nylon membrane as described previously [39]. The transferred RNAs were hybridized with both radiolabeled PSTVd dimeric and 5S RNA probes. The Northern gel blot hybridizations were performed as described previously [39].

## 3. Results

### 3.1. Role of RdR6 in N. benthamiana Plants’ Phenotype

**Figure 1:**
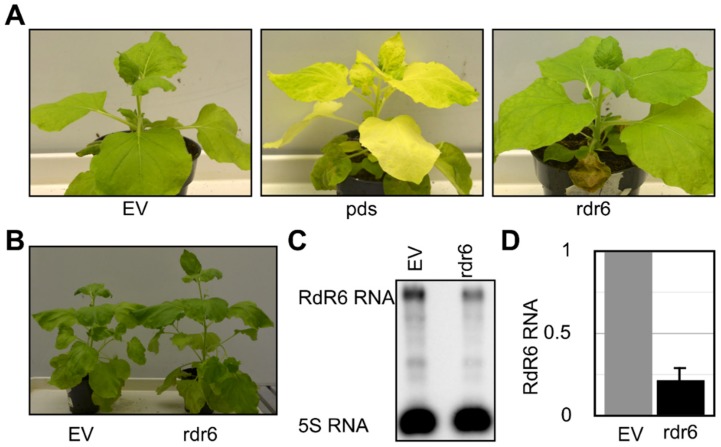
The knock-down of RdR6 in *N. benthamiana* resulted in a taller phenotype. *N. benthamiana* plants were subjected to a knock-down assay using a virus-induced gene silencing (VIGS) technique in order to verify the role of the RdR6 mRNA in determining the plant’s phenotype. (**A**) At 18 days post-agroinfiltration (dpa), the plants exhibited phenotypic alterations. (**B**) At 28-dpa the plants exhibited noticeable differences in their heights. Total RNA extracted from systemic leaf samples from the agroinfiltrated plants was subjected to (**C**) an RNA gel blot and (**D**) an RT-qPCR assay in order to evaluate the down-regulation of RdR6 in *N. benthamiana* plants. The expression change is presented on a log2 scale. EV, plants agroinfiltrated with pTRV2-EV vector; pds, plants agroinfiltrated with pTRV2:PDS vector; rdr6, plants agroinfiltrated with pTRV2-RdR6 vector. Each experiment was performed at least three times with true biological replicates.

In order to evaluate the role of RdR6 in the phenotype of *N. benthamiana*, its RdR6 gene was knocked-down by VIGS. The binary vectors pTRV2-RDR6 and pTRV2-PDS were agroinfiltrated into *N. benthamiana* seedlings, and the vector pTRV1 into the *Agrobacterium tumefaciens* strain GV3101, as described previously [40]. pTRV2-PDS, which is capable of down-regulating the *PDS* mRNA, was used as a positive control for the VIGS experiment as the knock-down of *PDS* mRNA results in bleached leaves. Negative control plants were maintained by agroinfiltrating the plants with pTRV2-EV along with pTRV1. At 21 days post agroinfiltration (dpa), the pTRV2-PDS plants exhibited bleached leaves (Figure 1A). Phenotypically, the plants agroinfiltrated with pTRV2-RdR6 looked very similar to the pTRV2-EV inoculated plants, except for their height (Figure 1A). At 28-dpa, the pTRV2-RdR6 agroinfiltrated plants exhibited a striking difference in their heights when compared to the pTRV2-EV inoculated plants (Figure 1B). The pTRV2-RdR6 agroinfiltrated plants were, on average, 9 cm (*n* = 10) taller than the pTRV2-EV agroinfiltrated plants at 28-dpa. The transgenic RdRi *N. benthamiana* developed by Schwach et al. (2005) did not exhibit any morphological changes as compared to the parental plants. Additionally, the seeds obtained from these plants were used by other researchers for viroid infection assays [32,33], but none observed the phenotypic changes reported here. The down-regulation of the expression of the RdR6 gene was confirmed by both RT-qPCR and RNA gel blot assay (Figure 1C). The data presented here demonstrate the involvement of RdR6 in the determination of the height of *N. benthamiana* plants.

### 3.2. The Suppression of RdR6 Increased PSTVd Accumulation in N. benthamiana

**Figure 2:**
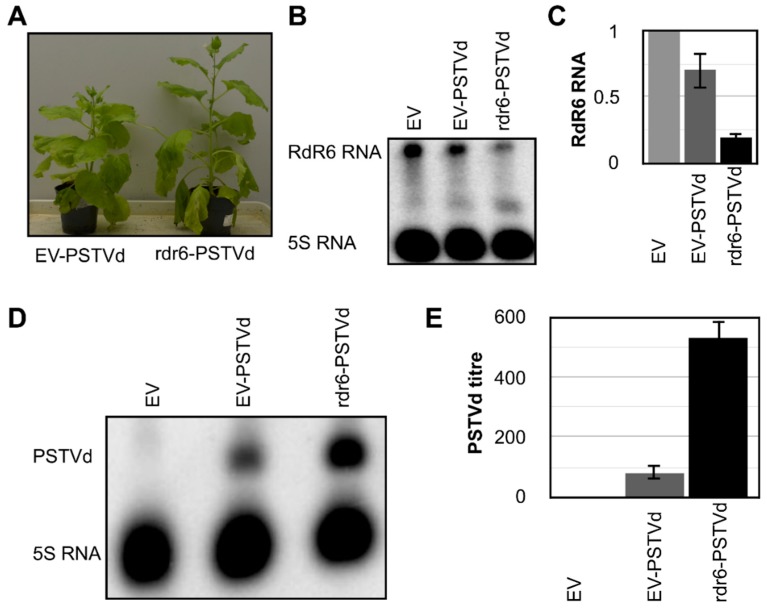
RdR6 compromised *N. benthamiana* plants exhibited higher amounts of PSTVd. The RdR6 knocked-down *N. benthamiana* plants were subjected to a PSTVd-RG1 infection assay in order to verify the role of the RdR6 mRNA in viroid accumulation. (**A**) At 21 days post-inoculation (dpi), the plants did not exhibit any viroid-associated disease symptoms. Total RNA extracted from *N. benthamiana* plants at 21 dpi was used to monitor the expression of RdR6 by (**B**) RNA gel blot and (**C**) RT-qPCR assay using RdR6 specific radiolabeled probes and primers, respectively. The same RNA sample was also used to evaluate the accumulation of PSTVd RNA by (**D**) RNA gel blot and (**E**) RT-qPCR assays using PSTVd specific radiolabeled probes and primers, respectively. In the (**E**) PSTVd titer represents the quantity of PSTVd RNA obtained by RT-qPCR. The expression change is presented on a log2 scale. The error bars indicate the standard deviation (SD). EV-PSTVd, plants agroinfiltrated with pTRV2-EV vector and infected with PSTVd-RG1; rdr6-PSTVd, plants agroinfiltrated with pTRV2-RdR6 vector and infected with PSTVd-RG1. Each experiment was performed at least three times with true biological replicates.

In order to evaluate the role of RdR6 in the defense against PSTVd, the PSTVd-RG1 variant was used in the infection assay. Upon infection, PSTVd-RG1 is known to induce severe symptoms in tomato plant cv. Rutgers. Although there were reports of PSTVd-RG1-induced symptoms in *N. benthamiana* [33], no PSTVd-induced symptoms in *N. benthamiana* plants were observed under the growth conditions used here. However, a faster accumulation of the PSTVd-RG1 variant in both *N. benthamiana* and tomato plants, as compared to that of both PSTVd-intermediate (PSTVd-I; GenBank Acc. No. AY937179) and PSTVd-Dahlia (PSTVd-D GenBank Acc. No. AB623143: the term PSTVd-M was used as this variant induces mild symptoms in tomato plants cv. Rutgers) was observed [35] (the data related to the *N. benthamiana* infection assay are not shown). In order to perform the bioassays, the RdR6 gene of the *N. benthamiana* plants was knocked-down as described before. Plants agroinfiltrated with pTRV2-PDS were used as VIGS controls, while the plants agroinfiltrated with pTRV2-EV were used as experimental controls. At approximately 18-dpa, that is to say, when pTRV2-PDS plants started to show bleaching, the plants agroinfiltrated with either pTRV2-RdR6 or pTRV2-EV were inoculated with the PSTVd-RG1 transcripts. These plants were named rdr6-PSTVd and EV-PSTVd, respectively. Mock-inoculated pTRV2-EV was used as the negative control (called EV-M, hereafter). At 21-dpi, neither the EV-PSTVd nor the rdr6-PSTVd plants, showed any viroid-induced symptoms. Interestingly, the rdr6-PSTVd plants were taller than either the pTRV2-EV or the M-EV plants, as was observed previously (Figure 2A). In order to monitor both the suppression of RdR6 expression and the accumulation of viroid, leaf samples collected at 21-dpi were used to extract total RNA. The suppression of RdR6 in the rdr6-PSTVd plants was evaluated by RNA gel blot assay using radiolabelled riboprobes specific for the RdR6 RNA and was compared to the results in both the EV-M and the EV-PSTVd plants (Figure 2B). Additionally, the suppression of RdR6 is further verified by RT-qPCR assay using RdR6 specific primers (Figure 2C). In order to evaluate the effect of RdR6 on the accumulation of the viroid RNA, the same RNA samples were subjected to RNA gel blot assays using radiolabelled dimeric (−) PSTVd riboprobe directed against the (+) PSTVd RNA strand. As shown in Figure 2D, rdr6-PSTVd plants exhibited increased accumulation of PSTVd-RG1 RNA as compared to the EV-PSTVd plants. In order to quantify the accumulation of PSTVd-RG1 RNA in the rdr6-PSTVd plants, RT-qPCR performed on the same RNA samples using PSTVd specific primers. As shown in Figure 2E, rdr6-PSTVd plants showed approximately a three-fold increase in PSTVd-RG1 when compared to that seen in the EV-PSTVd plants. Taken together, the data show that the suppression of RdR6 increases the susceptibility of the plant to viroid accumulation, and the involvement of RdR6 in the defensive pathway against PSTVd infection.

### 3.3. RdR6 Compromised Plants Exhibited Higher Amounts of PSTVd-sRNA

**Figure 3:**
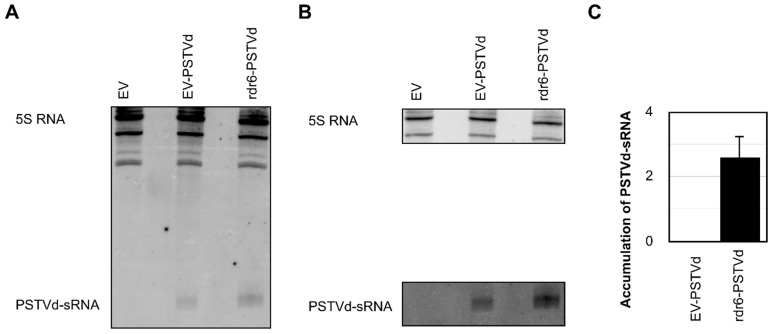
Differential PSTVd-sRNA accumulation in *N. benthamiana* plants. (**A**) Total RNA extracted from *N. benthamiana* plants at 21-dpi was used to monitor the PSTVd-sRNA levels by RNA gel blot assay using a radiolabeled PSTVd riboprobe. The 5S rRNA was used as a loading control. (**B**) A different contrast than was used in (**A**) so as to permit better visualization of the pertinent bands. (**C**) The gel blot signals were quantified and expressed as a ratio of the PSTVd-sRNA to the 5S rRNA signals. For each set of experiments, the ratios of target RNA to 5S rRNA obtained with EV-PSTVd inoculated plants were set at a value of zero. The error bars indicate the standard deviations (SD). EV-PSTVd, plants agroinfiltrated with pTRV2-EV vector and infected with PSTVd-RG1; rdr6-PSTVd, plants agroinfiltrated with pTRV2-RdR6 vector and infected with PSTVd-RG1. Each experiment was performed at least three times with true biological replicates.

Upon infection, viroids are known to trigger the RNA silencing machinery, and the presence of such vd-sRNAs has been reported by many researchers using various viroid-host combinations [7,8]. As RdR6 is known to be involved in the production of sRNA through the RNA silencing machinery, it would be interesting to verify the production/accumulation of vd-sRNA in RdR6 compromised plants. Hence, the total RNAs isolated from EV-M, EV-PSTVd, and rdr6-PSTVd plants at 21-dpi were examined by fractioning the sRNAs by denaturing (8M urea) polyacrylamide gel electrophoresis followed by RNA gel blot hybridization using radiolabelled dimeric (−) PSTVd riboprobes. Interestingly, the rdr6-PSTVd plants exhibited more vd-sRNA than did the EV-PSTVd plants (Figure 3A,B), specifically at least 2.5-fold more (Figure 3C). The accumulation of higher amounts of vd-sRNA in rdr6-PSTVd indicated either the involvement of another RdR molecule or that the small quantity of vd-sRNA processed by RdR6 follows another host defense machinery in order to enhance vd-sRNA production.

## 4. Discussion

Viroids are non-coding circular, RNA pathogens discovered in the early 1970s [40]. Despite their lack of any protein-coding capacity [41], studies have shown that viroid infection triggers the host’s RNA silencing machinery and induces viroid-associated symptoms through the generation of vd-sRNA [9,10,35]. Previously, the role of RdR6 in viroid-induced symptom expression was described using an *N. benthamiana*-hop stunt viroid combination [32]. Recently two independent studies revealed the possibility of the vd-sRNA independent degradation of host mRNAs during viroid infection [14,15]. These two studies indicated the possibility of the involvement of RdR6 in transgene silencing during viroid infection. In order to verify the role of RdR6 on both viroid accumulation and vd-sRNA production, the RdR6 of *N. benthamiana* plants was suppressed by VIGS. Though Schwach et al., [23] have already developed transgenic RdR6 knock-down plants that have been used by a couple of groups in order to study the role of RdR6 during viroid infection, it was decided to use the transient expression of RdR6 for the knock-down assay because during the process of producing the transgenic plants the host’s genome is modified by the insertion of a known gene sequence (example, RdR6) into an unknown position of the host’s genome. Hence, the TRV based knock-down of RDR6 was used, as the transient expression of RdR6 will not modify any of the host’s genes. Interestingly, under the experimental conditions used here, the RdR6 compromised *N. benthamiana* plants grew taller than the control plants (Figure 1). The transgenic RdRi *N. benthamiana* developed by Schwach et al., [23] did not exhibit any morphological changes when compared to the parental plants. Additionally, the seeds obtained from these plants were used by other researchers for viroid infection assays [32,33], but none observed any phenotypic changes such as were seen here. However, RdR6 has been shown to be involved in a vegetative phase change in *Arabidopsis,* as RdR6 mutant lines resulted in minor phenotypes [42]. The *Arabidopsis* mutants of *RdR6* showed an earlier transition from the juvenile vegetative to adult vegetative phase as compared to that of wild-type plants [43]. Additionally, RdR6 mutants also demonstrated an up-regulation in the transcription factor SPL3. In *Arabidopsis*, SPLs proteins are important positive effectors of vegetative phase change, and their expression temporally increases during development [44]. This could explain the phenotypic changes observed in RdR6 suppressed *N. benthamiana* plants in this study.

Since the RdR6 compromised plants showed higher susceptibilities to certain viruses, it was expected that the PSTVd bioassay with RdR6 compromised plants would show a higher accumulation of viroid molecules. Quantitative analysis by RT-qPCR revealed an approximately three-fold increase in the accumulation of viroid molecules as compared to that seen in the EV-PSTVd plants (Figure 2). These findings are in agreement with a previous report in which RdR6 mutant *N. benthamiana* lines showed increased levels of PSTVd-NB (GenBank Acc. No. AJ634596.1) accumulation [33]. As RdR6 plays a major role in the host’s defense system against RNA pathogens, the reduction of RdR6 activity might have increased the host’s susceptibility to PSTVd infection. Although RdR6 plays a major role in transgene silencing by converting single-stranded RNA into double-stranded RNA [45], a recent study showed that RdR6 is required for the efficient hairpin RNA induced RNAi seen in plants [46]. In other words, as viroids are highly structured molecules, and they fold into a hairpin RNA structure, the presence of RdR6 is essential for the efficient silencing of these viroid molecules. Hence, in the absence of RdR6, a plant is more vulnerable to viroid infection, thus leading to the greater accumulation of viroid molecules.

Subsequently, an RNA gel blot assay revealed at least a 2.5-fold higher vd-sRNA accumulation level in RdR6 compromised *N. benthamiana* plants than in EV-PSTVd plants (Figure 3). The increase in vd-sRNA seen in RdR6 knocked-down plants is in agreement with a previous study [33]. Although this increase in accumulation correlates with the proportion of viroid molecules detected by RT-qPCR, it should be noted that RdR6 is required for the efficient silencing of a viroid which structurally behaves as a hairpin RNA molecule (for detailed structures see references [1,47]). In this scenario, one could speculate on either the possible involvement of another RdR, or that of a different defense pathway. Recent studies on the deep sequencing data of siRNAs of viral origin established that RdR1 and RdR6 function synergistically during virus infection through RNA silencing [48]. At the same time, it could also be wise to think that the primary vd-sRNA produced by PSTVd in the absence of RdR6 may not be good regulators of RNA interference. Interestingly, the time-course analysis of viroid infection in transgenic RdRi plants revealed a decrease in viroid titer after a certain period of infection [33]. Although no time-course viroid accumulation assays were performed in this study, we previously observed that the PSTVd titer decreased after certain weeks of infection depending on the variant [35]. Furthermore, Sano et al., [49] noted a decrease in the PSTVd RNA concentration during the late stage of infection which was visualized by diminishment in the disease symptoms in the late stages of infection that was called “recovery phenomenon”. This could be explained by a multi-faceted research project which focuses on viroid population dynamics, its effect on RdR6 and other plant defense genes which play crucial roles in viroid infection and proliferation.

The data presented here demonstrate that RdR6 plays a role in the host’s phenotype, at least in *N. benthamiana*. Additionally, the absence or the suppression of RdR6 leads to the higher accumulation of PSTVd in *N. benthamiana* plants, indicating the increased susceptibility of the host to viroid infection. Although the RdR6 compromised plants showed a higher accumulation of vd-sRNA, due to the absence of RdR6 that is required in order to produce secondary sRNA a the more efficient anti-viroid defense, these plants showed a high accumulation level of mature PSTVd RNA. Clearly, further research is required in order to shed light on the mechanism underlying this anti-viroid defense, as well as on the production of secondary RNAs in the presence of RDR6 during viroid infection.
